# Programmed intermittent epidural bolus versus continuous epidural infusion for postoperative analgesia after major abdominal and gynecological cancer surgery: a randomized, triple-blinded clinical trial

**DOI:** 10.1186/s12871-018-0613-6

**Published:** 2018-10-30

**Authors:** Thomas Wiesmann, Lilli Hoff, Lara Prien, Alexander Torossian, Leopold Eberhart, Hinnerk Wulf, Carsten Feldmann

**Affiliations:** Department of Anesthesiology and Intensive Care Medicine, Philipps University Marburg, University Hospital Marburg (UKGM - Campus Marburg), Baldinger Strasse, 35033 Marburg, Germany

## Abstract

**Background:**

Continuous epidural infusion (CEI) is the standard application setting for epidural infusion. A new mode, the programmed intermittent epidural bolus (PIEB) technique, showed reduced local anesthetic (LA) consumption and improved analgesia in obstetric analgesia. Goal of this trial was to evaluate the effects of PIEB versus CEI [combined with patient-controlled bolus (PCEA)] on LA consumption and pain scorings in major abdominal cancer surgery.

**Methods:**

Following ethical approval, patients scheduled for major abdominal cancer surgery under general anesthesia in combination with epidural analgesia were randomized to receive either a PIEB mode of 6 mL/h or a CEI mode set at 6 mL/h of ropivacaine 0.2%, both combined with a PCEA mode set at a 4 mL bolus. LA consumptions and pain scorings were documented until the second postoperative evening.

**Results:**

Eighty-four datasets were analyzed (CEI: *n* = 40, PIEB: *n* = 44). Regarding the primary endpoint, cumulative LA PCEA bolus volumes until day 2 differed significantly between the groups [PIEB 10 mL (2–28 mL) versus CEI, 28 mL (12–64 mL), median (25th–75th percentiles), *p* = 0.002]. Overall, LA consumption volumes were significantly lower in the PIEB group versus in the CEI group [PIEB: 329 mL (291–341 mL) vs. CEI: 350 mL (327–381 mL), *p* = 0.003]. Pain scores were comparable at each time point.

**Conclusions:**

This trial demonstrates reduced needs for PCEA bolus in the PIEB group. There were no clinically relevant benefits regarding morphine consumption, pain scorings, or other secondary outcome parameters.

**Trial registration:**

This study has been registered retrospectively in the ClinicalTrials.gov registry (NCT03378804), date of registration: December, 20th 2017.

**Electronic supplementary material:**

The online version of this article (10.1186/s12871-018-0613-6) contains supplementary material, which is available to authorized users.

## Background

Major abdominal cancer surgery is regularly linked with intermediate to severe pain following the procedure. Postoperatively, analgesic therapy should be performed by a multimodal combination of systemic analgesics and the use of regional anesthesia techniques such as thoracic epidural analgesia [1, 2]. Additional proven effects of epidural analgesia such as reduced time to the recovery of bowel function are beneficial after abdominal surgery [3]. Epidural infusion is usually administered by an elastomeric or electronic pump delivering continuous local anesthetic (LA) flow via the indwelling epidural catheter, in addition to patient-controlled epidural bolus (PCEA) applications. Recent advances in infusion technology have resulted in the technique of a programmed intermittent epidural bolus (PIEB) at a set interval [4]. In obstetric analgesia, this application mode prompted a significant decrease of LA consumption, [4] potentially due to a better distribution of LA in the epidural space in comparison with the continuous infusion technique. However, this technique has not yet been evaluated for major abdominal surgery.

To our knowledge, this is the first randomized triple-blinded trial (patient blind, researcher blind, blinded statistician) to investigate the effects of PIEB versus continuous epidural infusion (CEI) for postoperative analgesia in combination with PCEA option after major open abdominal and gynecological (i.e., pancreatic, colonic, ovarian, and cervical) cancer surgery on LA consumption (primary endpoint) and postoperative pain management including opioid consumption and potential side effects (secondary endpoints).

## Methods

### Ethics, consent & permissions

Following approval by the local ethics committee (Ethical Commission, University Hospital Marburg, AZ 119/16) and registration in the ClinicalTrials.gov registry (NCT03378804), this triple-blinded, randomized, and controlled single-center study was performed in accordance with the Declaration of Helsinki. Written informed consent was obtained from all participating subjects prior to enrollment. This manuscript adheres to the applicable CONSORT guidelines.

Patients (ASA classification 1 to 4) scheduled for elective major abdominal (pancreatic and colonic) and gynecological (ovarian and cervical) cancer surgery with scheduled midline laparotomy were screened between January 2017 and November 2017. Exclusion criteria were age < 18 years or > 80 years, an inability to give consent, pregnancy, general contraindications for or an inability to undergo thoracic epidural analgesia techniques, inability to use a patient-controlled epidural analgesia (PCEA) technique, scheduled postoperative mechanical ventilation, and known or suspected allergy to LA.

Predefined dropout criteria were failed epidural anesthesia, revision surgery within the first 24 h and mechanical ventilation during intensive care unit stay.

### Anesthesia, surgery, and postoperative analgesia

In the induction area, standard monitoring was applied according to current national guidelines. Insertion of a thoracic epidural catheter (Perifix; B. Braun, Melsungen, Germany), using a loss-of-resistance technique was performed after skin disinfection in the sitting position at the Th8–Th9 or the Th9–Th10 interspaces under mild sedation using 5 μg to 15 μg of intravenous (IV) sufentanil. Following negative aspiration and negative response to a test dose of 3 mL of bupivacaine 0.5% (without epinephrine), the indwelling catheter was fixed using sterile drapings (Tegaderm; 3 M, Maplewood, MN, USA). An initial dose of 15 mL of ropivacaine 0.375% was applied via the indwelling epidural catheter with the patient in the supine position. After 15 min, cold/warm sensibility testing was performed bilaterally to evaluate the appropriate spread (multisegmental sensory blockade) of the epidural block.

Anesthesia induction was performed with sufentanil 0.2 μg kg^− 1^ to 0.3 μg kg^− 1^, propofol 2 mg kg^− 1^ to 3 mg kg^− 1^, and cisatracurium 0.1 mg kg^− 1^ or rocuronium 0.5 mg kg^− 1^ to 0.6 mg kg^− 1^. Intraoperatively, a balanced anesthesia technique using desflurane (target bispectral index values of 35 to 55) and remifentanil 0.1 μg kg^− 1^ min^− 1^ to 0.25 μg kg^− 1^ min^− 1^ was administered. Intraoperatively, all patients underwent active warming to achieve normothermia. All patients received a standardized IV double antiemetic prophylaxis using dexamethasone 4 mg to 8 mg and granisetrone 1 mg or droperidol 0.625 mg to 1.25 mg with regard to patient-specific conditions [5]. Recovery from neuromuscular blockade was monitored in all patients, a reversal was performed individually at the discretion of the respective anesthetist if the Train-of-Four (TOF) ratio did not reach 4/4 and the Double Burst Stimulation (DBS) showed a residual blockade phenomena. Prior to emergence from anesthesia at the end of the operation, all patients received 15 mg kg^− 1^ to 20 mg kg^− 1^ metamizole (dipyrone) as a nonopioid analgesic component of our standard multimodal analgesic regimen.

After surgery, patients were extubated in the operation room and transferred either to the postanesthesia care unit (PACU) or—if so scheduled—directly into the intensive care unit for further care.

### Intervention groups

At the beginning of the operation, a PCEA pump (AmbIT® PIB PCA; Teleflex, Wayne, PA, USA) with a sterile bag containing ropivacaine 0.2% (Naropin® 2%; AstraZeneca, Cambridge, UK) and sufentanil 0.75 μg/mL^− 1^ (Sufentanil Hameln, Hameln Pharma, Hameln, Germany) was connected to the epidural catheter. The PCEA pump was either programmed with a CEI of 6 mL/h flow or using the programmed intermittent bolus (PIB) mode with 6 mL bolus every 60 min according to the randomization result (details are given below). Both pump settings were combined with a patient-controlled bolus option (PCEA) of 4 mL (lockout time: 30 min). Staff members as well as patients were unaware of the randomization results until the time of overall data analysis.

### Postoperative analgesia

Staff nurses and patients were asked to use the PCEA whenever the numeric rating scale (NRS; range: 0–10) for pain at rest of the patient was 4 or higher. Patients were encouraged to use the PCEA during ward stay until postoperative day (POD) 3 whenever they were not receiving adequate pain control (NRS > 4). In the case of insufficient analgesic response to the PCEA bolus (to reach an NRS < 4 within 15–20 min after bolus application, IV rescue analgesia with the opioid piritramide (3.75–7.5 mg intravenously) was allowed as a ‘rescue option’. Patients received a combination of oral ibuprofen and metamizole (dipyrone) as part of our institutional multimodal protocol. In patients with repetitive needs for systemic opioids, 10 mg to 20 mg of prolonged-release oxycodone was given orally twice daily.

#### Sample size calculation

**Fig. 1:**
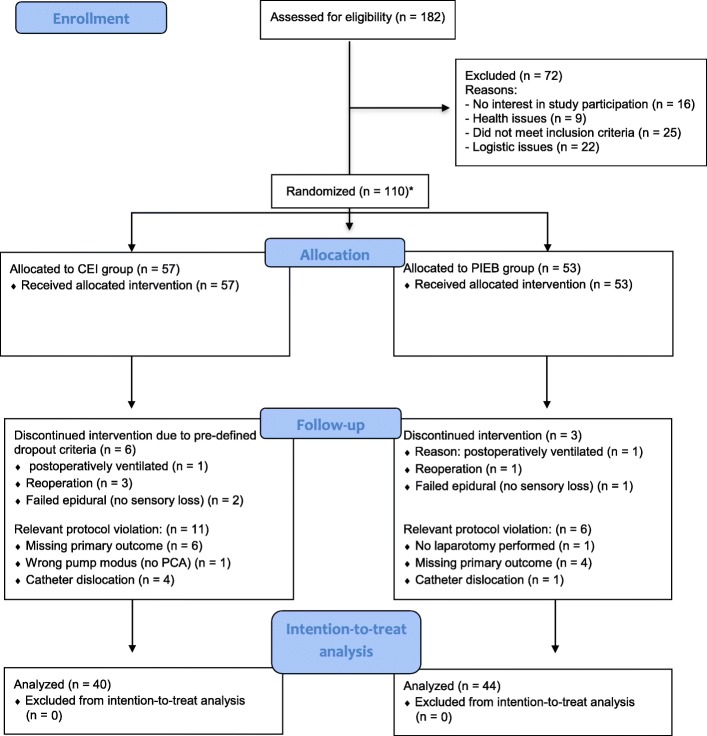
CONSORT Flowchart. *Initial, randomization of 90 patients was planned. During study period, further ethical permission was given for in total 110 patients due to an unexpected higher rate of drop-outs and protocol violations. For details, see text

Sample size calculation was performed according to data on LA consumption differences between CEI and PIB modes in obstetric anesthesia provided by a meta-analysis by George et al. [4] as well as our own historical patient data. Hourly LA consumption was calculated to equal 5 mL/h of ropivacaine 0.2% [standard deviation (SD): 1.7 mL/h] in the conventional group in contrast to the programmed intermittent epidural group, which was calculated to equal 4 mL/h ropivacaine 0.2% (SD: 1.35 mL/h). Given an alpha value of 0.05 and a beta value of 0.8, we calculated the minimum required sample size of patients per group to be 37 to detect a group difference of 1 mL/h assuming a standard deviation in the two groups of one third of their means (PASS 15; NCSS, Kaysville, UT, USA). This number was initially increased to 45 patients per group to compensate for dropouts. During the active study period, a relevant number of dropouts and serious protocol violations (which were not related to the study, e.g. reoperations) occurred (Fig. 1). Thus, after obtaining permission from the ethical commission, another 20 participants were recruited and randomized to achieve the relevant number of included patients for statistical analysis as determined by the sample size calculation. Hence, overall 110 patients were finally randomized.

#### Randomization, allocation, and blinding

A computer-generated sequence of random numbers was used to randomize the study participants with a 1:1 ratio of CEI to PIEB, both in combination with a PCB mode, using the specific modes of the PCEA pump system. To avoid imbalances due to the three different types of abdominal surgery (colonic/pancreatic/gynecological surgeries), a stratified randomization approach was used. The randomization sequence was generated by an investigator (LE) who had no further involvement in the patient treatment using the aforementioned Internet-based randomization tool (www.random.org) to generate a block randomized list (block size of 6 patients). The allocation was concealed in sealed, opaque, sequentially numbered envelopes. For each study participant, one envelope was handed to an assistant not involved with the study or a patient care assistant who was assigned to program the electronic pump for the epidural analgesia according to the randomization result. In a running pump LCD display, the overall amount of applied volume is shown but not the respective mode of delivery as programmed by the first, unblended operator. Neither the patients nor the responsible anesthetists or study assistants were aware of the randomization results. According to the triple-blinded study approach, unblinding was performed following completed statistical analysis of the blinded study allocation.

#### Primary and secondary endpoints

The primary endpoint of this trial was cumulative overall LA volumes of the PCB mode until 48 h after surgery. Secondary endpoints included delivered CEI or PIEB mode-derived volumes plus delivered PCB volumes (overall LA consumption) as well as the LA volumes per mode (CEI or PIEB) delivered at different time points until the evening of the operation day (POD0) and the first and second postoperative day (POD1–POD2).

Pain was evaluated using the NRS (0–10; 0, no pain; 10, worst imaginable pain) at rest, during cough, and during movement.

IV morphine equivalents were calculated for the first 3 days separately and overall. Oral and intravenously applied opioids other than IV morphine were converted into IV morphine equivalents (i.e., IV morphine 10 mg = oral morphine 30 mg = IV hydromorphone 1.5 mg = oral hydromorphone 7.5 mg = oral oxycodone 20 mg = IV tramadol 100 mg = IV piritramide 7.5 mg) according to the published literature [6].

Time to the first passage of flatus and time to the first passage of feces (stool) were calculated as the time of the end of the surgery until the first event of each, respectively. Nausea and vomiting were calculated based on dichotomous variables. Overall patient satisfaction was assessed using a numeric rating score from 0 to 10.

We assessed sensory spread (by using a standardized cold-warm testing method) bilaterally 15 min after the initial LA bolus application via the indwelling epidural catheter in the preoperative area as well as after surgery at POD0, POD1, and POD2. Patient-reported numbness was investigated as dichotomous variable (yes / no), signs of motor-blockade were investigated according to Bromage’s score as an ordinal-scaled variable from 0 to 4 (degrees of motor blockade: 0, none; 1, inability to perform hip flexion; 2, inability to perform knee flexion; 3, inability to move legs or feet).

Assessments were performed in a standardized fashion by one of the same two investigators (LH and LP) to reduce interobserver variability.

#### Statistical analysis

Continuous variables are presented in the format of the mean ± SD for normally distributed data or as medians (including the 25th–75th interquartile range) for data with non-normal distributed variables. Nominal and ordinal (categorical) variables are presented as n (%). The normality of distribution was tested using histograms and QQ plots as well as statistical analysis using the Shapiro–Wilk test. Modified intention-to-treat statistical analysis was performed for all analyzed parameters. Fisher’s exact test for categorical variables or a chi-squared test were applied when appropriate for testing differences in groups. An analysis of continuous variables was performed using the Student’s *t*-test for mean differences or the Mann–Whitney U test when nonparametric testing was necessary. Hodges–Lehmann estimators for the location of the shift parameter were given for local anesthetic consumption values for the evaluation of clinically relevant differences. Two-sided *p*-values were reported. Statistical significance was judged at a type I error level of 0.05. Statistical analysis was performed using SPSS (release 22; IBM Corp., Armonk, NY, USA).

## Results

From January 2017 until November 2017, 182 patients were assessed for eligibility. Of these, 72 patients did not participate in the study (16 patients refused to participate in the study, 25 patients did not meet inclusion criteria, nine patients had medical contraindications, 17 were excluded due to logistic issues such as postponed or cancelled surgery, and five were excluded due to other reasons). Finally, 110 patients were randomized (Fig. 1). Blinded intention-to-treat analysis of 84 patients was performed according to protocol. Details of dropout reasons and relevant protocol violations are given in Fig. 1.

Among the 84 patients analyzed (40 patients in the CEI group and 44 patients in the PIEB group), baseline patient characteristics were found to be similar between the groups (Table 1).

**Table 1:** Demographic and perioperative data

	Group CEI (*n* = 40)	Group PIEB (*n* = 44)	*p*-value
Age, y	58 ± 16	63 ± 12	0.09
Sex, (M/F)	9/31	15/29	0.24
Weight, kg	80 ± 21	79 ± 20	0.96
Height, cm	167 ± 8	168 ± 9	0.61
BMI, kg/m^2^	29 ± 7	28 ± 7	0.81
ASA status I/II/III	0/23/17	1/28/15)	0.37
Length of hospital stay, d	14 (10–18)	12 (10–16)	0.53
Type of surgery (colonic/pancreatic/gynaecological)	12/09/19	14/10/20	0.84
Length of surgery, min	205 (171–329)	173(135–253)	0.06
Epidural insertion height (lower spinal segment)	Th10 (Th9-Th11)	Th10 (Th9-Th11)	0.60
Epidural catheter performance time, min	12(8–17)	10(7–12)	0.14
Length of epidural catheter therapy, d	5 (5–7)	5 (5–6)	0.33
MAP initial (MAP1), mmHg	107 (91–123)	103 (93–114)	0.46
MAP at initial ropivacaine administration (MAP 2), mmHg	97 (90–116)	100 (89–113)	0.81
MAP 15min after initial ropivacaine administration (MAP 3), mmHg	88 (76–97)	86 (78–98)	0.94
Sufentanil for EDA placement, μg	0 (0–10)	0 (0–5)	0.24
Metamizole intraoperative, g	1.5 (1–1.5)	1.5 (1.5–1.5)	0.76
Metamizole d0, g	2 (1–3.5)	2 (1–3)	0.73
Metamizole d1, g	4 (4–4)	4 (3–4)	0.22
Metamizole d2, g	4 (4–4)	4 (4–4)	0.41
Ibuprofen d0, g	0.8 (0.4–0.8)	0.8 (0.6–0.8)	0.66
Ibuprofen d1, g	1.2 (0.8–1.4)	1.2 (0.6–1.2)	0.83
Ibuprofen d2, g	1.2 (1.2–1.6)	1.2 (1.2–1.8)	0.33
Opioids d0, % (yes/no)	30 (12/28)	36.4 (16/28)	0.54
Opioids d1, % (yes/no)	45 (18/22)	40.1 (18/26)	0.71
Opioids d2, % (yes/no)	67.5 (27/13)	50 (22/22)	0.11
Opiods d0–2, % (yes/no)	75 (30/10)	66 (29/15)	0.37

Values are expressed as the mean ± SD, median (25th–75th percentile), number of patients (n), or absolute numbers (%). *PIEB* programmed intermittent epidural bolus group, *CEI* continuous epidural infusion group, *BMI* body mass index, *ASA* American Society of Anesthesiology, *EDA* epidural anesthesia, *MAP* mean arterial pressure, *LA* local anaesthetic; Statistical significance was tested using Mann-Whitney-U-Testing, T-Testing or Chi-square where appropriate, two-sided *p*-value. Level of significance *p* < 0.05

### Local anesthetic consumption

Regarding the primary outcome measurement, the applied PCEA bolus volumes were significantly higher in the CEI group [28 mL (12–64 mL)] versus in the PIEB group [10 mL (2–28 ml), *p* = 0.004, Table 2]. Additionally, the differences between the need for additional PCEA bolus were statistically different on POD1 and POD2 but not so from the initial time of surgery until the evening of the same day (Table 2).

**Table 2:** Local anesthetic consumption

	Group CEI (*n* = 40)	Group PIEB (*n* = 44)	*p*-value	HL-Estimator (95% CI)
LA PCA Bolus d0-d2, ml	28 (12–64)	10 (2–28)	0.002*	16 (4; 28)
LA PIEB/CEI amount d0-d2, ml	323.5(286.6–336.7)	311.4 (263–330.2)	0.161	10.2 (−4; 30.6)
Overall LA consumption d0-d2, ml	350.3 (326.5–380.8)	329 (291–341.1)	0.003	29.55 (10.7;52.1)
LA PCA Bolus d0, ml	0 (0–8)	0 (0–4)	0.046	0 (0; 0)
LA PCA Bolus d1, ml	18 (4–26)	4 (0–14)	0.002*	8 (4; 16)
LA PCA Bolus d2, ml	12 (4–34)	4 (0–12)	0.010*	4 (0; 12)
LA PIEB/CEI amount d0, ml	50.9 (36–55.7)	48 (36–54)	0.238	2.9 (−1,8; 6.8)
LA PIEB/CEI amount d1, ml	140.9 (132.4–145.2)	132.3 (120–141.7)	0.015*	8.3 (1.2; 17.2)
LA PIEB/CEI amount d2, ml	135.2 (110.6–141.4)	131 (109.5–140.1)	0.588	2.3 (−6.6; 11)

*LA* local anesthestic, *d0* day of operation; *d1/d2* first/second postoperative day; *PIEB* programmed intermittent epidural bolus, *CEI* continuous epidural infusion, *PCA* patient controlled bolus, *HL* Hodges-Lehman. Values are expressed as the median (25th–75th percentiles), Mann-Whitney-U-Testing, two-sided p-value. Uncorrected *p*-values are displayed. Level of significance *p* < 0.05. *, significant

A statistically significant difference in overall LA consumption was observed in the PIEB group [329 mL (291–341.1 mL); median 25th–75th percentiles] as compared with in the CEI group [350.3 mL (326.5–380.8 mL), *p* = 0.01] until 48 h postoperatively (POD2). The LA volumes applied by the CEI or PIEB mode alone (without PCEA bolus volumes) were not significantly different between the CEI group [323.5 mL (286.6–336.7 mL)] and the PIEB group [311.4 mL (263–330.2 mL), *p* = 0.161].

#### Pain scorings and analgesic therapy

NRS value at rest, during cough, and during movement did not differ significantly between the CEI and PIEB groups for each given time point (Table 3). The amounts of ibuprofen and metamizole given as part of our multimodal regimen also did not differ between the groups for the given time points (Table 1). Additionally, overall, the need for opioids (as calculated as IV morphine equivalents) was not significantly different in the CEI group [15 mg [2.5–22.5 mg]] versus in the PIEB group [7.5 mg (0–20 mg), *p* = 0.27] between the end time of the surgery and POD2.

**Table 3:** Pain scoring

	Group CEI (*n* = 40)	Group PIEB (*n* = 44)	*p*-value
NRS rest d0	0 (0–0)	0 (0–0)	0.21
NRS rest d1	0 (0–3)	0 (0–2.8)	0.94
NRS rest d2	0.5 (0–3)	0 (0–2.5)	0.34
NRS cough d0	0 (0–3)	0 (0–0)	0.06
NRS cough d1	3 (1–6)	3 (0–5)	0.49
NRS cough d2	2.5 (0.5–5.5)	2.5 (0–6)	0.81
NRS movement d0	0 (0–3)	0 (0–0)	0.32
NRS movement d1	3 (1–5.5)	3 (0–4.5)	0.39
NRS movement d2	3 (0–5)	2 (0–4.5)	0.41
Morphine IV d0, mg	5 (5–7.5)	5 (5–5)	0.03
Morphine IV d1, mg	10 (10–10)	10 (10–10)	0.53
Morphine IV d2, mg	10 (10–15)	10 (10–10)	0.94
Morphine IV d0-d2, mg	15 (2.5–22.5)	7.5 (0–20)	0.27
Opioid need d0, yes / no	12/28	16/28	0.54
Opioid need d1, yes / no	18/22	18/26	0.71
Opioid need d2, yes / no	27/13	22/22	0.11
Opioid need d0-d2, yes / no	30/10	29/15	0.37

Values are expressed as the median (25th–75th percentile and as two-sided p-value of the Mann-Whitney –U test. Uncorrected *p*-values are displayed. Level of significance *p* < 0.05. No significant differences were found after Bonferroni correction. *NRS* numeric rating scale, *d0–3* postoperative day 0–3, *LA* local anaesthetic

### Side effects

Patients in both groups had low needs of necessary norepinephrine therapy in the evening after surgery (3/46 in the CEI group vs. 3/44 in the PIEB group, Table 4). On POD1, one patient in the CEI group and two patients in the PIEB group, respectively, required norepinephrine, whereas, on POD2, no patients required additional norepinephrine.

**Table 4:** Side effects / Clinical parameters

	Group CEI (*n* = 40)	Group PIEB (*n* = 44)	*p*-value
Vasopressor need d0, % (yes/no)	7.5 (3/37)	6.8 (3/41)	0.88
Vasopressor need d1, % (yes/no)	2.5 (1/39)	4.5 (2/42)	0.62
Vasopressor need d2, % (yes/no)	0 (0/40)	0 (0/44)	1.00
Nausea d0, % (yes/no)	20 (8/32)	18.2 (8/36)	0.79
Nausea d1, % (yes/no)	22.5 (9/31)	25 (11/33)	0.79
Nausea d2, % (yes/no)	32.5 (13/27)	13.6 (6/38)	0.04
Vomiting d0, % (yes/no)	17.5 (7/33)	11.4 (5/39)	0.40
Vomiting d1, % (yes/no)	20 (8/32)	22.7 (10/34)	0.76
Vomiting d2, % (yes/no)	17.5 (7/33)	15.9 (7/37)	0.85
Satisfaction d0, NRS	10 (10–10)	10 (10–10)	0.15
Satisfaction d1, NRS	10 (10–10)	10 (9–10)	0.19
Satisfaction d2, NRS	10 (8–10)	10 (8–10)	0.84
EDA catheter removal, day	5 (5–7)	5 (5–6)	0.33
Time to first flatus, hours	22.8 (18.6–43.8)	23.1(20.3–45.5)	0.25
Time to first defecation, hours	71.2 (40.8–119)	65.8(32.4–93.5)	0.54

Values are expressed as the median (25th–75th percentiles) or numbers (percents) and as two-sided p-value of the Mann-Whitney –U test or Fisher’s exact test when appropriate. Uncorrected p-values are displayed. Level of significance *p* < 0.05. *NRS* numeric rating scale, *d0–3* postoperative day 0–3, *LA* local anaesthetic, *EDA* epidural anesthesia

Time to first passage of flatus was not significantly different in the CEI group [22.8 h (17.6–42.2 h)] as compared with in the PIEB group [23.1 h (20.3–45.5 h), *p* = 0.25]. The time to first passage of feces after surgery was also not significantly different between the CEI group [71.2 h (40.8–119 h)] and the PIEB group [65.8 h (32.4–93.5 h), *p* = 0.54]. Frequencies of nausea and vomiting were not significantly different between groups at the evening of the operation, the first and the second postoperative day. Patient satisfaction at d1 and d2 was not significantly different between groups (Table 4). Additional information regarding sensory spread and motor blockade testing is given in the Additional file 1.

## Discussion

To our knowledge, this is the first randomized trial that has investigated different modes of epidural local anesthetic application (continuous vs. programmed intermittent technique) in patients undergoing major abdominal cancer surgery. Our study shows a statistically significant decrease of administered ropivacaine 0.2% until POD2 in patients who experienced PIEB as compared with CEI, both in combination with a patient controlled bolus (PCB) option. Overall pain scores did not differ significantly between the groups at rest, during cough, or during mobilization for each given time point until POD2. Additionally, overall postoperative morphine consumption showed comparable low values. Time to first passage of flatus or feces (stool) were not significantly different between the groups.

### Analgesic effects and morphine consumption

Our study results are consistent with previously published obstetric data showing reduced local anesthetic consumption when using a PIEB technique versus a traditional CEI [4, 7–10]. However, the overall reduction of LA consumption between the day of operation and POD2 was approximately 15 mL, resulting in questionable clinical relevance. Again, this is in line with previous reports that showed only mild reductions in LA volumes when the PIEB technique was used. On the other hand, this significant difference is explainable by a reduction of applied PCEA bolus volume as a surrogate of better analgesia. This effect of reduced PCEA bolus consumption in the PIEB group as compared with in the CEI group was consistently shown on POD1 and POD2 but not on the day of the surgery. This may be the result of the large initial bolus of 15 mL of ropivacaine 0.375% that was used for improved sensory testing quality of the epidural technique in the induction area prior to surgery. Pain scores were comparable between the groups, which is explainable by the fact that IV opioids were given as a rescue option in addition to the patient controlled bolus application option of the epidural infusion pump.

### Limitations

Our study has several limitations and strengths that need to be addressed.

First, there was a relevant number of dropouts and protocol violations (26 of 110 randomized patients). This was the result of several relevant factors: specifically, reoperations due to early complications as well as unplanned prolonged mechanical ventilation with necessary sedation were classified as severe protocol violations as they potentially interfered with the primary and secondary endpoints of our study. Thus, we decided to exclude patients who experienced these scenarios from our analysis. Additionally, the decision to choose the local anesthetic consumptions as endpoints were problematic in our clinical practice, as some staff members accidentally switched empty LA bags of our study patients and cleared the pump history, which resulted in the loss of the endpoint parameters in several patients.

As a second limitation, the decision to choose local anesthetic consumption as a primary endpoint might be questioned by some colleagues. This surrogate endpoint was selected for use in our study as previous investigations (mainly in obstetric populations) showed no differences in pain scores or rescue opioid use between CEI and PIEB modes. Moreover, from an ethical point of view, it would be inhuman to expose one trial group to higher pain scores by denying rescue opioids just to aim for a difference in pain scorings. Even a difference of 10 mm in the 100 mm visual analogue scale for pain was noted by patients as a relevant difference in pain according to the results of a recent study by Myles et al [11].

Lastly, the optimal PIEB mode has yet to be determined with regard to programmed bolus volume, time interval, local anesthetic, or its concentration [8, 12, 13]. Thus, we chose a pragmatic approach using our standard settings for epidural catheters of 6 mL/h^− 1^ for the CEI group and the same volume for the PIEB in the PIEB group. We did not perform an economic analysis of the different types of epidural infusion. PIEB modes are restricted to specific types of commercially available electronic pumps, which potentially might result in higher costs in comparison with other pump systems. Moreover, the programmed intermittent bolus application is applied by the pumps with a higher pressure than in continuous infusion technique, resulting in higher energy consumption as compared with the continuous infusion [14]. When using alkaline batteries, a shorter interval of battery exchange might have a considerable economic and ecologic impact [15]. Thus, if available, A/C adapters or rechargeable batteries should be considered when using PIEB modes, regardless of the specific manufacturer.

## Conclusion

In this triple-blinded randomized trial, local anesthetic consumption was significantly lower in patients with a PIEB versus a standard CEI. However, overall pain scores were comparable, as were side effects and patient satisfaction scorings. From a clinical perspective, the overall reduction of local anesthetic consumption was mild comparing the PIEB mode with the CEI mode. Hence, the use of PIEB mode in thoracic epidural analgesia after major abdominal surgery should be further evaluated with regard to optimized programmed bolus volumes and time intervals and outcome before widespread implementation in the clinical setting.

## Additional file

Additional file 1: Sensory & motor blockade testing. (DOCX 104 kb)

## Abbreviations

ASA: American Society of Anesthesiology
CEI: Continuous epidural application
d: Day
EDA: Epidural anesthesia
LA: Local anesthetics
MAP: Mean arterial pressure
ml: Millilitres
NRS: Numeric rating scale
PACU: Postoperative care unit
PCB: Patient controlled bolus
PCEA: Patient controlled epidural analgesia
PIEB: Programmed intermittent epidural bolus
POD: Postoperative day
SD: Standard deviation
