# Supplementary material for: Programmed intermittent epidural bolus versus continuous epidural infusion for postoperative analgesia after major abdominal and gynecological cancer surgery: a randomized, triple-blinded clinical trial
Source: BMC Anesthesiol. 2018 Oct 30;18:154. doi: 10.1186/s12871-018-0613-6 (PMC6208106; doi:10.1186/s12871-018-0613-6)
Supplement: Supplementary file 1 — Sensory & motor blockade testing. (DOCX 104 kb) [file 12871_2018_613_MOESM1_ESM.docx]

| **Table 5. Sensory & motor blockade testing** | | | |
| --- | --- | --- | --- |
|  | **Group CEI (n = 40)** | **Group PIEB (n = 44)** | **p-value** |
| Sensory spread left preop, no. of dermatomes | 19 (14-24) | 18 (13-21) | 0.44 |
| Sensory spread right preop, no. of dermatomes | 19 (14-25) | 18 (14-20) | 0.34 |
| Sensory spread left preop, cranial dermatome | C5 (C3-Th5) | Th2 (C5-Th4) | 0.19 |
| Sensory spread right preop, cranial dermatome | Th1 (C4-Th5) | Th3 (C5-Th4) | 0.20 |
| Sensory spread left preop, caudal dermatome | L4 (L2-S5) | S5 (L3-S5) | 0.34 |
| Sensory spread right preop, caudal dermatome | S5 (L2-S5) | S5 (L3-S5) | 0.81 |
| Sensory spread left d0, no. of dermatomes | 17 (8-20) | 19 (14-21)’ | 0.035’ |
| Sensory spread right d0, no. of dermatomes | 14 (10-22) | 19 (13-21) | 0.22 |
| Sensory spread left d1, no. of dermatomes | 5 (0-9) | 9 (0-12) | 0.06 |
| Sensory spread right d1, no. of dermatomes | 6 (0-11) | 8 (0-13) | 0.23 |
| Sensory spread left d2, no. of dermatomes | 0 (0-2) | 4 (0-9) | 0.024’ |
| Sensory spread right d2, no. of dermatomes | 0 (0-6) | 0 (0-9) | 0.53 |
| Numbness d0, yes/no | 8/32 | 12/32 | 0.48 |
| Numbness d1, yes/no | 7/33 | 11/32 | 0.38 |
| Numbness d2, yes /no | 4/36 | 9/35 | 0.19 |
| Motor Blockade left, d0, Grade 0/1/2 | 1/39 | 4/40 | 0.23 |
| Motor Blockade right, d0, Grade 0/1/2 | 1/39 | 5/39 | 0.14 |
| Motor Blockade left, d1, Grade 0/1/2 | 0/40 | 1/43 | 0.34 |
| Motor Blockade right, d1, Grade 0/1/2 | 0/40 | 1/43 | 0.18 |
| Motor Blockade left, d2, Grade 0/1/2 | 0/40 | 1/43 | 0.34 |
| Motor Blockade right, d2, Grade 0/1/2 | 0/40 | 1/43 | 0.34 |
| Data are expressed as the median (25th-75th percentile) and as two-sided p-value of the Wilcoxon-Mann-Whitney test. d0-2, day of operation (d0) and first and second postoperative day. Degrees of motor blockade: 0, none; 1, inability to perform hip flexion; 2, inability to perform knee flexion) . Uncorrected p-values are displayed. Level of significance p < 0.05. ‘ , not significant after Bonferroni correction. | | | |
